# Attachment style, emotional feedback, and neural processing: investigating the influence of attachment on the P200 and P400 components of event-related potentials

**DOI:** 10.3389/fnhum.2023.1249978

**Published:** 2023-08-31

**Authors:** Inon Zuckerman, Ilan Laufer, Dor Mizrahi

**Affiliations:** Department of Industrial Engineering and Management, Ariel University, Ariel, Israel

**Keywords:** attachment style, emotional processing, feedback valence, event-related potentials, P200, P400, emotion regulation

## Abstract

Understanding the interplay between attachment style, emotional processing, and neural responses is crucial for comprehending the diverse ways individuals function socially and emotionally. While previous research has contributed to our knowledge of how attachment style influences emotional processing, there is still a gap in the literature when it comes to investigating emotional feedback using event-related potentials (ERPs) within a cognitive framework. This study aims to address this gap by examining the effects of attachment style and feedback valence on ERP components, specifically focusing on the P200 and P400. The findings reveal significant effects of attachment style and feedback valence on both components. In insecure attachment styles, noticeable shifts in relative energy are observed during the transition from negative to positive feedback for both the P200 and P400. Conversely, individuals with secure attachment styles exhibit minimal to moderate variations in relative energy, consistently maintaining a lower P200 energy level. Additionally, both secure and insecure individuals demonstrate heightened intensity in the P400 component in response to positive feedback. These findings underscore the influential role of attachment style in shaping emotional reactivity and regulation, emphasizing the significance of attachment theory in understanding individual differences in social and emotional functioning. This study provides novel insights into the neural mechanisms underlying the influence of attachment style on emotional processing within the context of cognitive task performance. Future research should consider diverse participant samples, employ objective measures of attachment, and utilize longitudinal designs to further explore the neural processes associated with attachment.

## 1. Introduction

Understanding the intricate relationship between attachment style, emotional processing, and neural responses is crucial for comprehending variations in social and emotional functioning among individuals (Bowlby, 1969; Mikulincer et al., 2009). Previous research indicates that individuals with insecure attachment tend to display heightened emotional reactivity and encounter challenges in regulating their emotions when compared to those with secure attachment (Mikulincer et al., 2009). Specifically, anxious and avoidant attachment styles have been linked to increased emotional sensitivity, stronger negative emotional reactions, and diminished emotion regulation abilities (Mikulincer et al., 2003; Vrtička et al., 2012).

Existing studies have demonstrated that attachment styles impact emotional responses (Rognoni et al., 2008) and neural activity during social perception (Chavis and Kisley, 2012). However, the specific role of attachment style in the interplay between cognitive processing and emotional feedback has not been thoroughly investigated. Some studies have examined the relationship between attachment dimensions and brain activation during gaze interactions (Cecchini et al., 2015), as well as the connection between attachment classifications and frontal EEG asymmetry (Gander and Buchheim, 2015). Other research has explored the response of individuals with fearful-avoidant attachment orientation to emotional stimuli (Dan et al., 2020) and the relationship between defense mechanisms, attachment, and depression (Békés et al., 2021). Additionally, studies have investigated the neural correlates of attachment dimensions during Rorschach inkblot tests (Lai et al., 2022) and the association between attachment insecurity and the α-EEG anomaly during sleep (Sloan et al., 2007).

Thus, there is a notable research gap in the existing literature regarding the specific examination of the role of attachment style in the interaction between cognitive processing and emotional feedback. In our study, we aim to fill this gap by employing ERPs to investigate how attachment style influences emotional responses. Specifically, we will analyze electrophysiological indices, such as the P200 and P400 components, to capture precise neural markers associated with cognitive and emotional processes. By integrating attachment theory with ERPs, we can gain a deeper understanding of how attachment style shapes emotional responses within the cognitive processing framework (Mikulincer et al., 2003; Vrtička et al., 2012).

The P200 component typically observed around 200 ms after stimulus presentation, is associated with early attentional processes and has been linked to the evaluation of emotional stimuli (Carretié et al., 2001). It reflects the initial orienting of attention toward emotionally salient information and can provide insights into the early perceptual processing of emotional cues. The P400 component, occurring approximately 400 ms after stimulus onset, is thought to reflect cognitive evaluation and elaborative processing of emotional stimuli (Dien et al., 2010). It is sensitive to the motivational and affective significance of stimuli and has been linked to processes such as the evaluation of emotional meaning and contextual integration of emotional information (Kanske and Kotz, 2007). In the context of emotional feedback, the P400 component can capture the cognitive and emotional processing associated with the evaluation of the feedback’s relevance and implications.

Furthermore, we consider the findings of Etkin et al. (2011), which challenge the traditional dichotomy of the medial prefrontal cortex (mPFC) and anterior cingulate cortex (ACC) into cognitive and affective subdivisions. According to their research on negative emotions, including fear and anxiety, both dorsal-caudal and ventral-rostral regions of the ACC and mPFC contribute to emotional processing. Dorsal-caudal regions are involved in appraising and expressing negative emotions, while ventral-rostral regions regulate emotional responses generated by limbic regions. These insights support our investigation into the role of attachment style in cognitive-emotional interactions.

Additionally, previous research has shown that individuals high in avoidance, an insecure attachment style, may exhibit reduced Error-Related Negativity (ERN), which is associated with response-error monitoring (Simon-Dack et al., 2021). However, when it comes to emotional processing and cognitive engagement, we hypothesize that individuals with insecure attachment styles will exhibit larger P200 and P400 responses to emotional feedback compared to those with secure attachment. The rationale behind this hypothesis is that insecure individuals, due to their heightened emotional reactivity and difficulties in emotion regulation, may exhibit enhanced attentional processes (reflected in the P200) and heightened cognitive evaluation and elaborative processing of emotional stimuli (reflected in the P400). In contrast, individuals exhibiting secure attachment styles are anticipated to demonstrate regulated P200 and P400 responses, indicating effective emotion regulation strategies and an enhanced capacity to process emotional feedback within a cognitive framework.

Importantly, the observed reduction in ERN among insecure individuals (Simon-Dack et al., 2021) specifically pertains to error processing, while the expectation of larger P200 and P400 responses in insecure individuals relates to their processing of emotional feedback. These variations in neural responses underscore the intricate relationship between attachment style and the diverse cognitive and emotional processes at play. Attaining a comprehensive understanding of the precise neural mechanisms associated with attachment style and emotional feedback within a cognitive context can offer valuable insights into individual differences in emotional reactivity and regulation. Moreover, such knowledge holds potential for informing clinical interventions and therapeutic approaches aimed at fostering emotional wellbeing and nurturing positive interpersonal relationships.

In conclusion, our study addresses a significant gap in the existing literature by examining the influence of attachment style on the interplay between emotional feedback and cognitive processing. By focusing on the P200 and P400 components of ERPs, we aim to uncover the differential neural responses of secure and insecure individuals to emotional feedback within a cognitive framework. Through this investigation, we seek to shed light on the underlying mechanisms through which attachment style influences emotional processing and contribute to a more comprehensive understanding of the complex dynamics between attachment, cognitive processes, and emotional experiences.

## 2. Materials and methods

### 2.1. Study stages and task

The study consisted of two phases. Initially, a group of 96 fourth-year engineering students. Participants (46 females) were right-handed with no neurological symptoms between the ages of 20 and 35 [years] (mean = 24.25 [years], SD = 2.0673), recruited on campus.

**FIGURE 1:**
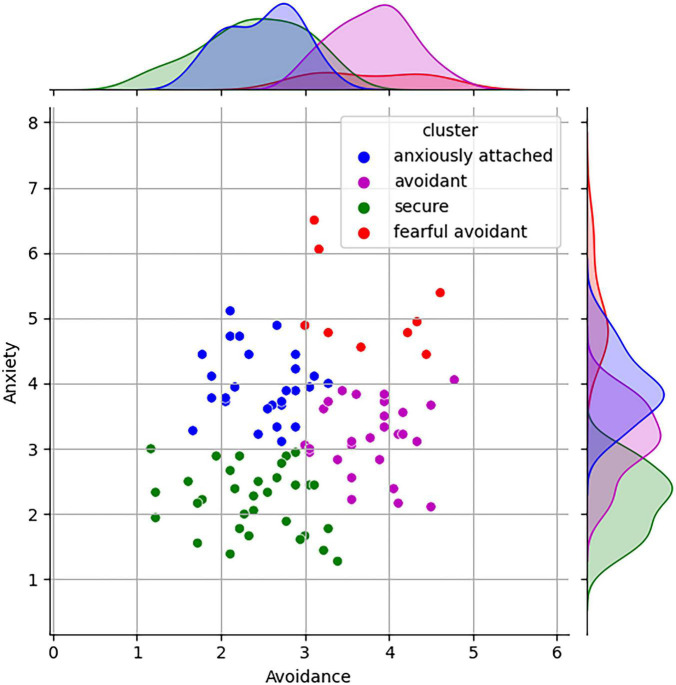
Clustered attachment results of the ECR-R questionnaire (*K* = 4).

The participants completed the ECR-R (Sibley and Liu, 2004; Sibley et al., 2005) questionnaire to assess their attachment styles. The ECR-R questionnaire is a widely used self-report instrument to measure adult attachment styles. It consists of 36 items grouped into anxiety and avoidance subscales. The anxiety subscale assesses fear of abandonment and desire for closeness, while the avoidance subscale evaluates discomfort with intimacy and preference for autonomy. It has strong psychometric properties and is valuable for understanding emotional and interpersonal functioning in relationships. Utilizing the k-means clustering algorithm, the data was classified into four attachment classes: secure, anxiously attached, avoidant, and fearful avoidant, which aligns with established literature (Chen et al., 2020) and presented in Figure 1. The centroid values of each attachment cluster are presented to provide a summary of their respective characteristics (Table 1).

**TABLE 1:** Attachment k-means clusters centroid.

Cluster	Avoidance cluster value	Anxiety cluster value
Secure	2.385	2.210
Anxiously attached	2.488	3.967
Avoidant	3.799	3.180
Fearful avoidant	3.759	5.148

In the subsequent phase, EEG recording sessions were conducted with a sample of 27 participants from the secure and insecure groups (anxiously attached, avoidant, and fearful avoidant). The mean age of the secure group was 23.66 [years] (SD = 1.50), the mean age of the insecure group was 23.85 [years] (SD = 2.53). The secure group comprised 6 participants, while the insecure group included participants from the anxiously attached, avoidant, and fearful avoidant classes (21 participants: 9 anxiously attached, 7 avoidant, and 5 fearful avoidant).

To achieve a comprehensive and unbiased distribution among various attachment clusters, we wanted to ensure that each cluster is adequately represented. This entails selecting participants in a manner that accounts for the diversity and proportions of individuals belonging to different attachment clusters, thereby facilitating a more accurate and balanced analysis of the data. To ensures a representative distribution across attachment clusters, a proportional allocation method was employed. This method involved selecting participants in a manner that maintained proportions consistent with the relative sizes of each attachment cluster (see Figure 1).

The decision to reduce the participants from the original four attachment style groups (anxious, secure, avoidant, and fearful avoidant) to two groups (secure and insecure) was motivated by the specific research focus of the study. The primary objective was to examine the differences between individuals with secure attachment styles and those with insecure attachment styles, rather than exploring the distinctions among the various insecure attachment subgroups. This approach enhances the statistical power and clarity of the results, as it allows for a more straightforward analysis and interpretation of the findings related to the central research question. Furthermore, reducing the number of groups to two simplifies the comparisons and avoids potential complexities arising from multiple group comparisons, which could increase the risk of Type I errors or require larger sample sizes to maintain adequate statistical power.

**FIGURE 2:**
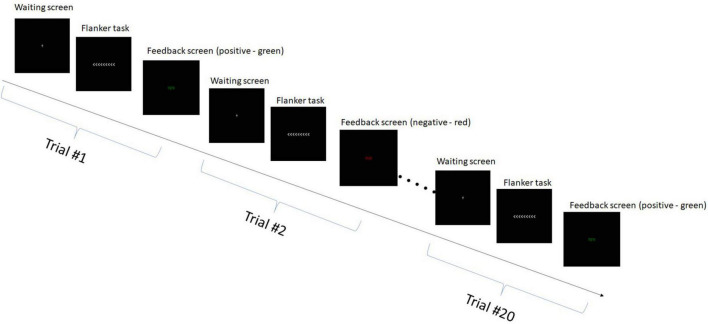
Experimental paradigm—single block. Each participant completed three such blocks.

During the EEG sessions, the participants engaged in the flanker task (Brunetti et al., 2019). This task involved the presentation of various arrow flanker configurations for 1 s, requiring participants to identify the direction of a central arrow amidst non-target stimuli and respond accordingly using the keyboard. The main objective of the flanker task was to measure the ability of participants to resist interference from the incongruent flanking arrows and maintain their attention on the central target. This required strong cognitive control and inhibitory processes to filter out the distracting information from the surrounding arrows. Each participant completed 60 trials of the flanker task, divided into three blocks of 20 trials each. There was a 1-min break between each block. The experimental paradigm is presented in Figure 2.

During the flanker task, participants responded to arrows presented on the screen by pressing the corresponding arrow key. In the first and third blocks, they matched the direction of the target arrow, while in the second block, they pressed the opposite direction. Feedback was given after each trial through color changes on subsequent slides, with correct trials shown as “correct” in green, and incorrect trials as “incorrect” in red for 1 s. Between trials, a gray cross was displayed on a black screen, and participants fixated on it for a randomly varied duration between 0.5 and 1.5 s. Each trial duration was ∼3 [sec], thus the total duration of a block comprising 20 tasks was ∼60 [s]. Prior to the main task, participants underwent a training session to become familiar with the experimental procedure.

### 2.2. EEG recordings

During EEG recordings, signals were recorded simultaneously with task performance using a 16-channel active EEG amplifier (e.g., USBAMP, by g.tec, Austria) operating at a sampling frequency of 512 Hz, adhering to the 10–20 international system. The electrode impedance was consistently maintained below 5 Kohm throughout the experiment, monitored by the OpenVibe (Renard et al., 2010) processing and recording software. A total of 1,600 epochs were collected, with 20 epochs excluded from the dataset due to poor-quality recordings from one participant. The data analysis in this study focused on six specific frontal and prefrontal electrodes, as previous research has consistently shown that increased activity in these regions correlates with enhanced cognitive abilities (Duncan and Owen, 2000; Miller and Cohen, 2001; Koechlin and Hyafil, 2007; Fuster, 2015; Banich, 2018). The prefrontal-frontal electrode cluster encompassed Fp1, F7, Fp2, F8, F3, and F7.

### 2.3. EEG pre-processing

The preprocessing pipeline began by applying a bandpass filter (1–30 Hz) to the continuous EEG data to remove unwanted frequency components and reduce noise. Next, independent component analysis (ICA) was performed on the filtered data to identify and separate independent components representing neural activity and artifacts. This approach ensured the removal of artifacts from the entire continuous dataset, maintaining data integrity. Following the ICA step, the continuous filtered data were segmented into individual epochs of 1-s duration. Epoching allowed for the isolation and analysis of specific 1-s time intervals, aligning with the duration of the flanker task slide.

### 2.4. Statistical analysis

Statistical analysis was conducted to examine significant differences in the mean amplitude measures of the P200 and P400 ERP components across different experimental conditions. A repeated measure Welch ANOVA (Tomarken and Serlin, 1986) was performed to assess the main effects of attachment style (secure, insecure) and feedback valence (positive feedback, negative feedback) on P200 and P400 mean amplitude, as well as the potential interactions between these factors. Welch Two-Way ANOVA is a statistical method used to analyze data with two independent variables (factors) when the assumption of equal variances and sample sizes is violated. It is suitable for situations where there are unequal group variances and/or imbalanced sample sizes in both factors. Welch Two-Way ANOVA offers a robust and reliable alternative to traditional two-way ANOVA, providing more accurate results and valid statistical inferences in such non-ideal conditions.

**FIGURE 3:**
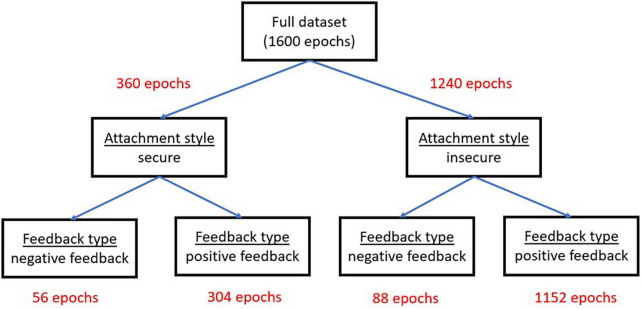
The distribution of the 1,600 EEG epochs per each factor level in the experimental mixed design.

Figure 3 presents the distribution of the entire dataset per each factor level.

## 3. Results

In all the subsections below, ERP components (P200 and P400) were analyzed using a Welch ANOVA with a 2 (Attachment Style: Secure vs. Insecure) × 2 (Feedback Valence: Positive vs. Negative) design.

### 3.1. Latency of first location of the maximum peak

**FIGURE 4:**
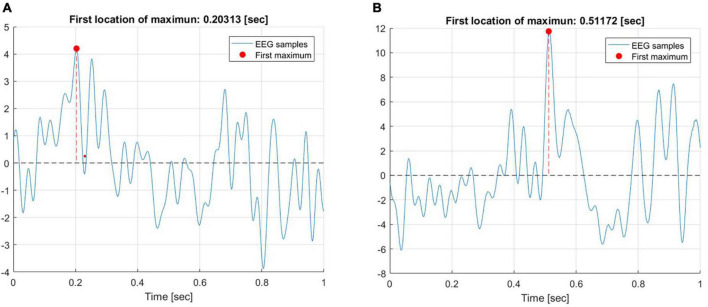
The maximum of the first peak in response to negative feedback for a representative participant from the insecure **(A)** and secure **(B)** groups.

Firstly, our objective was to investigate the prominent ERP peaks during the time interval of interest, specifically, within the first 1 s. epoch following the onset of the feedback slide. To that end, the latency of the first location of maximum amplitude of the average ERP trace was calculated for both the secure and insecure groups. The average trace was calculated across six frontal and prefrontal electrodes (Fp1, F7, Fp2, F8, F3, and F7). Figure 4 illustrates the maximum of the first peak in response to negative feedback for a representative participant from the insecure (A) and secure (B) groups, respectively.

**FIGURE 5:**
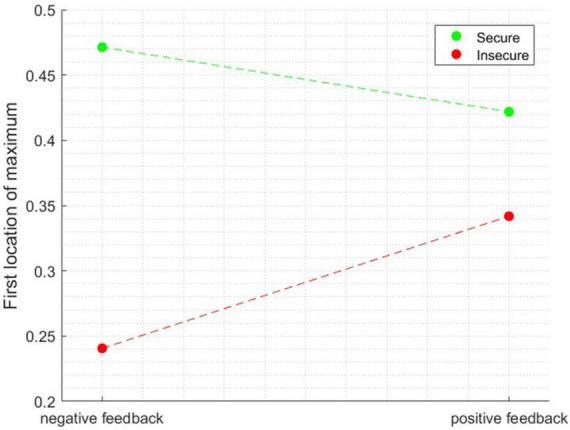
The interaction effect between the factors when the dependent variable is the latency of the first location of maximum peak.

The first peak for the insecure participant occurred around 203 ms, while the secure participant exhibited their first peak around 511 ms after feedback onset. It is noteworthy that this analysis was performed separately for each of the participants and the data was then entered into ANOVA. The ANOVA revealed a significant main effect of attachment style [*F*_(1, 1596)_ = 59.228, *p* < 0.0001] and a main effect of Attachment style × Feedback Valence interaction [*F*_(1, 1596)_ = 13.147, *p* < 0.001]. Figure 5 depicts the interaction between these two factors. The difference in the latencies of the first maximum peak was most pronounced for negative feedback and diminished for the positive feedback condition.

### 3.2. Relative energy computations

Next, we computed the relative energy (RE) of the intervals surrounding the P200 and P400 peaks. For the P200, the interval spanned from 170 to 230 ms, while for the P400, it ranged from 370 to 430 ms. With a sampling rate of 512 Hz, the P200 interval corresponded to samples 87 to 118, and the P400 interval corresponded to samples 189 to 220. Hence, the window length for each peak was equal to 32 samples (note that the entire signal length was 1,000 ms relative to feedback onset). To establish a baseline reference energy value, we assumed a uniform energy distribution across the entire signal. In this case, the baseline reference value is calculated as:

$$R\,E_{B\,a\,s\,e\,l\,i\,n\,e}=\frac{32}{512}=0.0625=6.25\%$$

#### 3.2.1. Relative energy computations for P200

**FIGURE 6:**
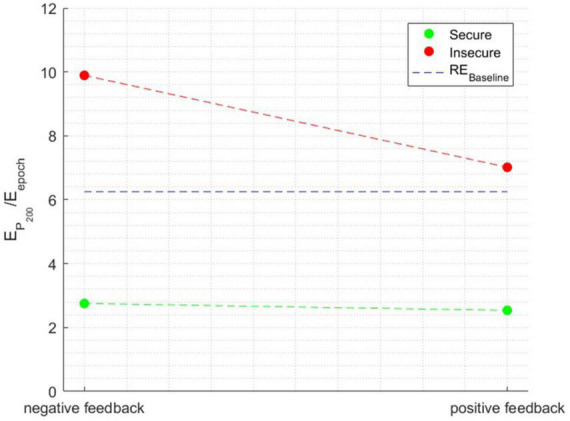
The interaction effect between the factors when the dependent variable is the relative energy in the time window surrounding the P200 peak.

The ANOVA performed with the relative energy in the P200 time window as the independent variable showed a significant main effect of attachment style [*F*_(1, 1596)_ = 225.1873, *p* < 0.0001], a main effect of Feedback Valence [*F*_(1, 1596)_ = 190.4927, *p* < 0.0001], and a main effect of Attachment style × Feedback Valence interaction [*F*_(1, 1596)_ = 8.4632, *p* < 0.001], η^2^=0.0722. Figure 6 illustrates the interaction effect between the factors when the relative energy of the time window surrounding the P200 peak was the independent variable.

Individuals with a secure attachment style exhibited minimal variation in relative energy between negative and positive feedback conditions. Conversely, individuals with insecure attachment displayed a significant change, with a noticeable decline in relative energy from negative to positive feedback, approaching the baseline level in the latter condition. Notably, secure individuals showed an extremely low energy level of only 2.5% in the P200 component, falling below the baseline.

#### 3.2.2. Relative energy computations for P400

**FIGURE 7:**
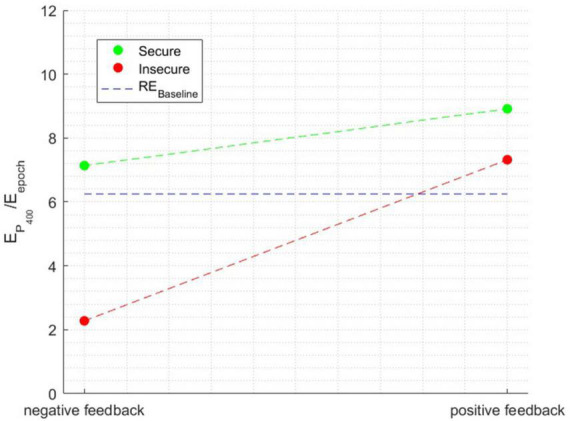
The interaction effect between the factors when the dependent variable is the relative energy in the time window surrounding the P400 peak.

The ANOVA performed with the relative energy in the P400 time window as the independent variable showed a significant main effect of attachment style [*F*_(1, 1596)_ = 128.7231, *p* < 0.0001], a main effect of Feedback Valence [*F*_(1, 1596)_ = 174.2652, *p* < 0.0001], and a main effect of Attachment style × Feedback Valence interaction [*F*_(1, 1596)_ = 51.671, *p* < 0.0001], η^2^=0.090. Figure 7 illustrates the interaction effect between the factors when the relative energy of the time window surrounding the P400 peak was the independent variable.

Both secure and insecure individuals displayed an increase in the intensity of the P400 component when receiving positive feedback, indicating a consistent trend across both groups. However, a notable distinction emerges in the response of secure individuals, as the decrease in the P400 component is comparatively more moderate. This disparity arises because secure individuals exhibit a significant P400 component in both positive and negative feedback conditions, with values surpassing the baseline. In contrast, insecure individuals demonstrate a significant component only in response to positive feedback.

In summary, for the P200 component, the study found significant effects of attachment style and feedback valence. Insecure individuals exhibited substantial changes in relative energy from negative to positive feedback, approaching baseline levels. In contrast, secure individuals displayed minimal variation in relative energy and maintained a lower overall P200 energy level.

Regarding the P400 component, both attachment style and feedback valence had significant impacts. In response to positive feedback, both secure and insecure individuals demonstrated increased intensity in the P400 component. However, secure individuals exhibited a more moderate decrease in response to negative feedback compared to insecure individuals, indicating a differential processing pattern between the two groups.

## 4. Discussion

The primary objective of this study was to investigate how attachment style and feedback valence impact ERP components, specifically the P200 and P400. Our hypotheses were partially supported, as we observed significant effects of both attachment style and feedback valence on these components. Insecure individuals displayed noticeable changes in relative energy when transitioning from negative to positive feedback for both the P200 and P400 (Pan et al., 2019). Conversely, secure individuals demonstrated minimal to moderate variations in relative energy and maintained consistently lower levels of P200 energy. Numerous studies have indicated that individuals who feel secure exhibit reduced amygdala reactivity when exposed to negative or threatening emotional stimuli. This diminished amygdala response suggests that secure individuals may possess a more adaptable and regulated style of emotional processing (Gillath et al., 2005; Dannlowski et al., 2011; Vrtička et al., 2012). Secure individuals may also demonstrate enhanced functional connectivity between prefrontal and limbic regions involved in emotional processing, such as the amygdala and hippocampus. This heightened connectivity could facilitate the top-down regulation of emotions and foster a more balanced and adaptive emotional response (Tidwell et al., 1996; Coan et al., 2006; Kim et al., 2014).

Our findings show that insecure attachment is associated with heightened emotional reactivity, whereas secure attachment is characterized by effective strategies for regulating emotions. Our study’s examination of neural responses reveals distinct differences that shed light on how attachment style influences emotional processing. Specifically, the processing patterns associated with negative feedback demonstrate notable distinctions in both the P200 and P400 components. In the case of insecure individuals, a pronounced P200 response to negative feedback indicates heightened sensitivity to negative emotional stimuli. This heightened response suggests that insecure individuals are more attuned to negative feedback and may experience stronger negative emotional reactions. It is possible that their increased vigilance for potential threats, coupled with a tendency to interpret negative feedback as personally meaningful or impactful, contribute to this heightened sensitivity (Meyer et al., 1999; Liu et al., 2011; Weinberg et al., 2012).

Conversely, secure individuals exhibit a regulated P200 response to negative feedback, indicating effective emotion regulation strategies or a greater ability to reinterpret negative feedback in a less threatening manner. The significantly lower amplitude of the P200 response observed in our study highlights their emotional resilience and adaptive coping mechanisms associated with secure attachment. Secure individuals may employ strategies such as reappraisal or cognitive reframing to maintain emotional stability despite negative feedback (Wei et al., 2005; Coan et al., 2006).

In addition to the P200 component, the P400 component also demonstrates differential patterns in response to feedback valence based on attachment style. Both secure and insecure individuals showed increased intensity in the P400 component for positive feedback (Gentzler et al., 2010). However, insecure individuals display a significant decrease in the P400 component in response to negative feedback, reflecting a heightened negative emotional response. This suggests that they are more sensitive to negative feedback compared to secure individuals. The pronounced P400 response in insecure individuals aligns with their tendency to perceive negative feedback as more threatening or personally meaningful, leading to a stronger emotional reaction. On the other hand, secure individuals exhibit a more moderate decrease in the P400 component, indicating regulated emotional responses even in the face of negative feedback. This finding underscores the emotional resilience and adaptive coping mechanisms associated with secure attachment (Karreman and Vingerhoets, 2012; Kural and Kovacs, 2021).

Secure individuals tend to exhibit increased activation in prefrontal regions of the brain, such as the dorsolateral prefrontal cortex (DLPFC) and the ventromedial prefrontal cortex (vmPFC), during the processing of emotional stimuli which may explain the more moderate decrease in the P400 component in response to negative feedback. These regions are associated with cognitive control, emotion regulation, and the integration of emotional and cognitive information. Eppinger et al. (2009) examined developmental differences in learning and error processing using event-related potentials (ERPs), including the P400 component. The results suggested that the DLPFC is involved in the regulation of cognitive control processes, specifically in the late positive component (LPC), which overlaps with the P400 component. These findings highlight the role of attachment style in modulating early neural processing of negative feedback, with insecure individuals showing heightened sensitivity in both P200 and P400 responses, possibly due to increased vigilance for threats and a tendency to perceive negative feedback as personally meaningful. In contrast, secure individuals display more regulated responses in both P200 and P400 components, indicating emotional resilience and effective emotion regulation strategies. This unravels how attachment-related factors influence emotional reactivity and regulation, shedding light on their connection to defense mechanisms.

The results of this study have important implications for clinical interventions and therapeutic approaches aimed at improving emotional wellbeing and interpersonal relationships. For example, brain-computer interface (BCI) and biofeedback techniques can focus on enhancing emotional regulation, increasing defense mechanism awareness, and utilizing electrophysiological markers such as P200 and P400 responses to guide treatment progress (Lim et al., 2012; Friedrich et al., 2014; Kural and Kovacs, 2021). Future research should explore the association between attachment and defense mechanisms in clinical or treatment samples to provide a more comprehensive understanding of the implications for psychopathology and therapeutic interventions (Ciocca et al., 2020; Békés et al., 2021). Understanding the influence of attachment style on neural processing can inform the development of tailored interventions that address individuals’ attachment-related needs. By targeting specific neural mechanisms involved in emotional processing, interventions can potentially enhance emotional regulation skills and promote more secure attachment styles.

Despite the insights provided by this study, several limitations should be acknowledged. The study focused on a specific age group and may not generalize to other populations or developmental stages both cognitively and emotionally. Our caution about generalizability pertains to individuals outside the young adulthood age range and encompasses various cognitive and emotional developmental stages that may exhibit different attachment patterns and neural processing of emotional information.

Self-report measures were used to assess attachment style, which can introduce biases and limitations. Future research should include more diverse samples to account for different cognitive and emotional stages such as adolescence, middle adulthood, or late adulthood. Moreover it is needed to utilize objective measures of attachment, and consider longitudinal designs to examine the stability and developmental trajectories of attachment-related neural processes. Moreover, it is important to consider the relatively small sample size of the attachment classes. Therefore, conducting future research with a larger and more diverse participant pool would be beneficial in confirming and further enhancing the validity of our findings.

In conclusion, this study deepens our understanding of how attachment style influences defensive responses and the role of defense mechanisms as moderators of the attachment system. It contributes to our knowledge of the impact of attachment style and feedback valence on neural processing, specifically the P200 and P400 components. These findings underscore the significance of attachment style in shaping emotional reactivity and regulation, highlighting the relevance of attachment theory for comprehending individual differences in social and emotional functioning. Further research in this area will offer valuable insights into the intricate interplay among attachment, neural processes, and emotional experiences.

## Data availability statement

The raw data supporting the conclusions of this article will be made available by the authors, without undue reservation.

## Ethics statement

The studies involving humans were approved by the Ariel University IRB Committee. The studies were conducted in accordance with the local legislation and institutional requirements. The participants provided their written informed consent to participate in this study.

## Author contributions

DM responsible for visualization and implementation of supporting algorithms. IZ and IL supervised the research activity. All authors carried out the stages of conceptualization, design of methodology, data curation, formal analysis, data modeling, model validation, writing, drafting and editing, discussed the results, read and approved the final manuscript and are accountable for all aspects of the work, read, and agreed to the published version of the manuscript.

## Acknowledgements

The authors would like to acknowledge the assistance of OpenAI’s ChatGPT model, version 3.5, for proofreading and grammar corrections of this manuscript. It is emphasized that the ChatGPT tool was solely utilized for the purpose of enhancing the grammatical accuracy and readability of the text. The entirety of the manuscript’s content was originally conceived and authored by human contributors without the generative assistance of AI in the creation or modification of the academic content.
