# Corrigendum: Attachment style, emotional feedback, and neural processing: investigating the influence of attachment on the P200 and P400 components of event-related potentials

**DOI:** 10.3389/fnhum.2024.1406968

**Published:** 2024-04-05

**Authors:** Inon Zuckerman, Ilan Laufer, Dor Mizrahi

**Affiliations:** Department of Industrial Engineering and Management, Ariel University, Ariel, Israel

**Keywords:** attachment style, emotional processing, feedback valence, event-related potentials, P200, P400, emotion regulation

In the published article, in the References section, the reference “Banich, M. T. (2002). Cognitive neuroscience and neuroimaging: A tool for empirical philosophy. Philos. Sci. 69, 572–586. doi: 10.3758/s13428-021-01632-3” was inaccurately cited. This should have been correctly written as “Banich, M. T., and Compton, R. J. (2018). *Cognitive Neuroscience*. Cambridge, MA: Cambridge University Press.”

In the published article, in the References section, the reference “Fraedrich, E. M., Lakhlani, D., McArthur, B. A., and Badenoch, N. (2010). Attachment security and emotion regulation: The mediating role of emotional reactivity. J. Child Fam. Stud. 19, 654–661” was inaccurately cited. This should have been correctly written as “Wei, M., Vogel, D. L., Ku, T. Y., and Zakalik, R. A. (2005). Adult attachment, affect regulation, negative mood, and interpersonal problems: the mediating roles of emotional reactivity and emotional cutoff. *J. Couns. Psychol*. 52:14. doi: 10.1037/0022-0167.52.1.14”.

In the published article, in the References section, the reference “Hahn, A. C., DeBruine, L. M., and Jones, B. C. (2015). Attachment style moderates the relationship between attractiveness-related social influence and visual attention to attractive opposite-sex faces. Pers. Individ. Dif. 78, 123–128” was inaccurately cited. This should have been correctly written as “Tidwell, M. C. O., Reis, H. T., and Shaver, P. R. (1996). Attachment, attractiveness, and social interaction: a diary study. *J. Pers. Soc. Psychol*. 71:729. doi: 10.1037/0022-3514.71.4.729”.

In the published article, in the References section, the reference “Johnson, M. H., and Oliver, A. (2019). The P400 event-related potential component: An index of cognitive evaluation and elaborative processing in response to emotional stimuli. Int. J. Psychophysiol 14, 86–95” was inaccurately cited. This should have been written as “Dien, J., Michelson, C. A., and Franklin, M. S. (2010). Separating the visual sentence N400 effect from the P400 sequential expectancy effect: cognitive and neuroanatomical implications. *Brain Res*. 1355, 126–140. doi: 10.1016/j.brainres.2010.07.099”.

In the published article, in the References section, the reference “Karreman, A., and Vingerhoets, A. J. (2012). Attachment and well-being: The mediating role of emotion regulation and resilience. Pers. Individ. Dif. 53, 821–826. doi: 10.1080/09638237.2019.1644492” was inaccurately cited. This should have been correctly written as “Karreman, A., and Vingerhoets, A. J. (2012). Attachment and well-being: the mediating role of emotion regulation and resilience. *Pers. Individ. Dif*. 53, 821–826. doi: 10.1016/J.PAID.2012.06.014”.

In the published article, in the References section, the reference “Smith, A. B., Jones, C. D., Smith, B. C., and Johnson, E. F. (2010). The P200 component of the event-related potential and its relation to attention and emotional processing: A meta-analysis. Clin. Neurophysiol. 121, 464–472” was inaccurately cited. This should have been correctly written as “Carretié, L., Martín-Loeches, M., Hinojosa, J. A., and Mercado, F. (2001). Emotion and attention interaction studied through event-related potentials. *J. Cogn. Neurosci*. 13, 1109–1128. doi: 10.1162/089892901753294400”.

In the published article, in the References section, the reference “Smith, N. G., Alkozei, A., Bao, S., Smith, S. S., and Killgore, W. D. (2019). Insecure attachment style and increased neural response to positive feedback: An event-related potentials study. Soc. Cogn. Affect. Neurosci. 14, 537–545” was inaccurately cited. This should have been correctly written as “Gentzler, A. L., Kerns, K. A., and Keener, E. (2010). Emotional reactions and regulatory responses to negative and positive events: associations with attachment and gender. *Motiv. Emot*. 34, 78–92. doi: 10.1007/S11031-009-9149-X”.

In the published article, in the References section, the reference “Vrtička, P., Bondolfi, G., and Sander, D. (2019). The neural substrates of social emotion regulation: A fMRI study on emotion reappraisal and suppression in individuals with and without social anxiety disorder. Front. Hum. Neurosci. 13” was inaccurately cited. This should have been correctly written as “Vrtička, P., Bondolfi, G., Sander, D., and Vuilleumier, P. (2012). The neural substrates of social emotion perception and regulation are modulated by adult attachment style. *Soc. Neurosci*. 7, 473–493. doi: 10.1080/17470919.2011.647410”.

In the published article, the Acknowledgments section was erroneously omitted. The newly added Acknowledgments section appears below:

“The authors would like to acknowledge the assistance of OpenAI's ChatGPT model, version 3.5, for proofreading and grammar corrections of this manuscript. It is emphasized that the ChatGPT tool was solely utilized for the purpose of enhancing the grammatical accuracy and readability of the text. The entirety of the manuscript's content was originally conceived and authored by human contributors without the generative assistance of AI in the creation or modification of the academic content.”

The authors apologize for these errors and state that they does not change the scientific conclusions of the article in any way. The original article has been updated.

